# A Peer Health Educator Program for Breast Cancer Screening Promotion: Arabic, Chinese, South Asian, and Vietnamese Immigrant Women's Perspectives

**DOI:** 10.1155/2015/947245

**Published:** 2015-02-24

**Authors:** Joanne Crawford, Angela Frisina, Tricia Hack, Faye Parascandalo

**Affiliations:** ^1^Department of Nursing, Brock University, 500 Glenridge Avenue, St. Catharines, ON, Canada L2S 3A1; ^2^Public Health Services, Healthy Living Division, Chronic Disease Prevention, 1447 Upper Ottawa Street, Hamilton, ON, Canada L8W 3J6

## Abstract

This study explored Arabic, Chinese, South Asian, and Vietnamese immigrant women's experiences with a peer health educator program, a public health program that facilitated access to breast health information and mammography screening. Framed within critical social theory, this participatory action research project took place from July 2009 to January 2011. Ten focus groups and 14 individual interviews were conducted with 82 immigrant women 40 years of age and older. Qualitative methods were utilized. Thematic content analysis derived from grounded theory and other qualitative literature was employed to analyze data. Four dominant themes emerged: *Breast Cancer Prevention* focused on learning within the program, *Social Support* provided by the peer health educator and other women, *Screening Services Access for Women* centered on service provision, and *Program Enhancements* related to specific modifications required to meet the needs of immigrant women accessing the program. The findings provide insights into strategies used to promote breast health, mammography screening, and the improvement of public health programming. Perceived barriers that continue to persist are structural barriers, such as the provision of information on breast cancer and screening by family physicians. A future goal is to improve collaborations between public health and primary care to minimize this barrier.

## 1. Introduction

Breast cancer is the most common cancer diagnosed and the second leading cause of cancer death among North American women [1]. Early detection of breast cancer using mammography is an effective strategy that has the potential to reduce mortality and morbidity in average risk women 50 years of age and older [2]. Yet, mammography screening is not undertaken by all eligible women, and subgroups of women continue to be underscreened. Immigrant women who settle in westernized countries such as the United States (USA), United Kingdom (UK), and Canada have lower mammography screening than nonimmigrant native born women [3–5]. Despite unique differences in migration patterns and sociocultural context of settlement country, common barriers to breast cancer screening access exist. Individual-level barriers include fear of cancer, lack of English language proficiency, lack of knowledge, and transportation issues [6, 7]. System-level barriers include lack of local access, inconvenient hours of screening service operation, lack of health insurance, a male health care provider, and lack of recommendation by a physician [6–8]. Settlement neighbourhood and a lack of tailored approaches for outreach perpetuate barriers to screening for some groups of immigrant women [9].

One promising tailored approach to reduce barriers and increase rates to mammography screening is the use of peer health educators from the same culture of the target immigrant community [10]. A feasibility study of the North Carolina Breast Screening Program reported that black women felt the support received from lay health advisors encouraged them to consider or obtain mammography screening [11]. In a systematic review of US studies, pooled results from randomized controlled trials demonstrated stronger effects if the peer health educator was matched by race and ethnicity to the target population with statistically significant improvements in mammography screening (RR 1.58; 95% CI 1.29–1.93, *P* < 0.0001) [12].

The use of peer health educators is an individual-directed intervention. However, when this approach is combined with other access-enhancing strategies such as bilingual small media (pamphlet or brochures), arrangement of appointments, individual reminders, transportation, and accompaniment, screening uptake is more effective than individual-directed strategies alone [13]. Cancer screening uptake among Asian populations in the USA was improved as a result of utilizing multiple strategies that included peer health educators [14]. In another study, Latinos enrolled in a cardiovascular promotion program using lay health advisors reported increased awareness and motivation to incorporate and maintain positive health practices such as healthier foods or increased exercise into daily activities [15].

The peer health educator as an outreach strategy is more common among diverse ethnocultural groups; yet, there are limited studies that have explored immigrant women's experiences with a peer health educator program that utilized multiple strategies. The purpose of this paper is to report on the experiences of immigrant women who accessed one public health program that employed peer health educators and utilized additional access-enhancing strategies to promote health and cancer screening. In this program, peer health educators used social networks to promote outreach including attendance at multicultural or community events; culturally appropriate small media; and intermittent women's health sessions on healthy lifestyles, prevention, and cancer screening services. Additionally, they booked screening appointments, arranged transportation, facilitated reminder calls, and provided accompaniment to screening and follow-up. Peer health educators also supported immigrant women to access other health care and community services unrelated to screening. Public health nurses (PHNs) and four peer health educators on a chronic disease prevention team collaborated with cancer screening services and women's health services to facilitate access for immigrant women.

The main objective of this study was to explore Arabic, Chinese, South Asian, and Vietnamese immigrant women's experiences with a peer health educator program that reduced barriers and provided access to breast health information and mammography screening in a midsized urban city in Canada. Secondary objectives were to elicit immigrant women's recommendations on ways to improve the program and subsequent action to make changes to the program. This community-focused project was funded by the Canadian Breast Cancer Foundation over a period of two and a half years (July 2008–January 2011).

## 2. Materials and Methods

In this study, participatory action research (PAR) was utilized and underpinned by theoretical foundations of critical social theory drawing on critical social theorists, Freire [16] and Fals Borda [17]. PAR is congruent with the nature of public health work with immigrant communities, community health nursing, and collaborative community relationships. The peer health educator approach was based on Freire's [16] principle of education focused on the real life context of immigrant women and empowerment. Education delivered through the program aimed to (a) liberate immigrant women to reflect on healthy lifestyle behaviours; (b) identify issues within immigrant women's lives and discuss them with others; and (c) empower individuals to act by making changes.

In exploring immigrant women's experiences with the program, the PAR process facilitated dialogue between partners to address cancer screening; entailed reciprocal learning between PHNs and immigrant women; and promoted critical awareness, subjective reflection, shared power, and subsequent action [18]. The PAR team consisted of two PHNs coleading research activities, two PHNs supporting data collection, an immigrant women center coordinator, and five immigrant women facilitators. Immigrant women facilitators (IWFs) represented the four immigrant communities and thus had equal power on all decision-making processes and were remunerated for their time.

### 2.1. Ethical Approval

Research Ethics Board approval was received from an affiliate university prior to study commencement. Participants were invited to volunteer to participate in the study via a standardized telephone recruitment process.

### 2.2. Sampling

Purposive sampling was used to recruit participants from the peer health educator program database at the public health unit from January 2005 to December 2008. Criteria for inclusion were Arabic, Chinese, South Asian, and Vietnamese immigrant women; 40 years of age and older; and attendance at a women's health session or accompaniment to breast screening. Participants were contacted by bilingual IWFs via telephone who used a standard recruitment script. Full open disclosures of study purpose, procedures, risks, and benefits were provided during the initial contact, and verbal consent was obtained for those volunteering to attend a focus group. All women who expressed an interest in the study were supported to participate. Numerous immigrant women did not feel comfortable participating in a focus group setting and were offered one-on-one interviews.

### 2.3. Procedures

The study was conducted using focus group methods and one-on-one interviews. Focus groups are particularly useful in equalizing power among investigators and participants, enabling open dialogue between participants, and facilitating rich discussions [19]. The aim is to uncover shared and common knowledge providing greater insights of experiences with the peer health educator program. Also, the “cuing” phenomenon permits the exploration of multiple perspectives related to knowledge, thoughts, and experiences with the program. One-on-one semistructured interviews allowed women to share experiences in their own language, at a safe location, with an IWF from her community. These methods align with critical social theory and valuing of the diverse perspectives of immigrant women.

Focus groups were conducted by IWFs and cofacilitator volunteers of the same culture, both of which were native speakers of their respective language. The focus groups were conducted for each distinct cultural community using the preferred language, for example, Arabic, Chinese (Mandarin), South Asian (Urdu), and Vietnamese (Table 1). One-on-one interviews were conducted by IWFs. Settings of focus groups and interviews included immigrant women's centers, community organizations, and libraries, locations where women felt most comfortable. A semistructured interview guide developed using open-ended questions for focus groups and one-on-one interviews were reviewed within the team, modified, cross-culturally translated, and focus-tested with five immigrant women (Appendices A and B). Questions addressed the experience of immigrant women with the peer health educator program including learning about breast health and screening, the benefits and challenges of the experience, and strengths and areas of improvement. For women who participated in screening, additional questions focused on individual decision-making process, accompaniment, and the overall screening experience.

### 2.4. Rigor

To enhance rigor and completeness of data, focus groups and interviews were audiotaped with detailed notes taken by cofacilitators. An initial member check was undertaken after each focus group, whereby the moderator discussed key points with participants to obtain immediate feedback [20]. Also,* debriefing* was used directly after each focus group to discuss common and diverse findings, as well as focus group process issues. A second point of* member-checking* involved several team meetings held with IWFs to discuss preliminary categories and themes as a means to ensure data remained true to participant's experiences. The last and final method of* member-checking* was facilitated through three community forums to report back to participants and confirm themes and subthemes.

All data was transcribed verbatim into the language spoken in each group, translated into English and back-translated, and entered into NVivo version 8 (QRS). Data collection progressed until* saturation*, which was established when we were hearing the same themes repeated. Additionally, a colleague not directly involved in the project reviewed three transcripts to confirm emerging categories and themes. A reflective journal tracked team process, new learning, insights, and challenges. Sociodemographics of participants were captured for descriptive purposes.

### 2.5. Data Analysis

All transcribed and translated data was subjected to thematic content analysis using Burnard's [21] framework. Derived from grounded theory [22], content analysis literature [23], and other qualitative analysis techniques [24], it provided a systematic approach to open-coding, developing categories and making connections between themes and data collected within a comprehensive category system. This method provided a generic multistage process of analysis that included (a) reading through transcripts and notes thoroughly to gain an understanding of immigrant women's experiences as a whole; (b) rereading transcripts and notes and open-coding with researcher-developed categories generating as many categories as possible; (c) grouping categories into similar categories under broader themes; and (d) refinement of themes and subthemes [21]. Two PHNs with research expertise analyzed data independently and then came together to compare initial categories. Confirmability was facilitated by rereading transcripts and notes and ensuring categories and emergent themes captured the essence of the data. While the IWFs were not directly involved in data analysis due to individual time constraints or a lack of confidence or desire to be involved in this aspect of the research, they were consulted regularly during data analysis. As reported earlier, the IWFs also participated in team meetings to discuss preliminary findings and final themes so that we remained true to immigrant women's experiences.

## 3. Results

A total of 82 immigrant women participated in 10 focus groups and 14 semistructured one-on-one interviews undertaken from April 2009 to June 2009. Focus groups ranged from four to 10 participants. The majority of participants were Arabic and South Asian immigrant women. Two-thirds of participants were 50 years of age and older. The mean number of years in Canada was 11 years, with a range of <1 year to 36 years; over 50% of women resided in Canada for 10 years or less. The majority of immigrant women preferred to communicate in their native language (Table 2). Four dominant and interconnected themes emerged from the data.

### 3.1. Theme 1: Breast Cancer Prevention

This theme focused on immigrant women's perceptions of learning about breast health, risk reduction, breast cancer, and screening services. Three subthemes are presented.

#### 3.1.1. Learning about Health Behaviours

Of all immigrant women groups, Arabic women provided the greatest insights of the impact of learning from women's health sessions. For some, they embraced learning and took action to change lifestyle behaviours reinforcing their understanding of the link between health behaviours and the risk of breast cancer. South Asian, Chinese, and Vietnamese immigrant women mainly discussed learning the importance of screening from the program as a preventive behaviour.

*Yes, I totally changed some not so healthy ways I used to practice… Now my thinking has changed and I have become more knowledgeable about my breast's health and the quality of my food too.* (Interview 1, Arabic)

*From the health sessions, we learned that healthy food and exercise reduces the chance of having cancer.* (FG 3, Arabic, participant 1)

*People often think in this way, ‘I am perfectly fine right now, then why should I go for a test?' That is why this kind of program is so important in terms of changing people's mindset of developing an awareness of prevention in a pro-active way. So, I think it totally changed my mindset.* (Interview 1, Chinese)

*I can understand more about cancer, find out early, and can cure it early.* (FG 2, Vietnamese, participant 2)

#### 3.1.2. Self-Responsibility

Chinese and Arabic immigrant women were more empowered with knowledge gained from women's health sessions. They believed women had to assume individual responsibility to maintain good health. Arabic women felt that women needed to take advantage of learning opportunities within the program or elsewhere for themselves and to educate other women.

*Before when I heard that someone's got cancer, I just felt sorry for them but I didn't consider it something that could ever happen to me. But now after the program, I do feel that it is your own responsibility to care for your own body and health; no one can replace this role. You should care for yourself in daily life. It's not the doctor's sole responsibility to take care of you.* (Interview 1, Chinese)

*Because of this screening, I even put off my trip to China for next year; I don't want to miss it.* (Interview 1, Chinese)

*Now we become more active. We know a lot of things about our health and we keep following up for any new activities (new topic) in the program to teach ourselves and our friends….* (FG 3, Arabic, participant 2)

*I have become a strong thinker and educated and still need to know more about my health in general and about my breasts and breast cancer, especially if I am trying to teach other women in my community.* (Interview 2, Arabic)

#### 3.1.3. Hope

Across all immigrant women groups, learning about breast cancer and the rationale for screening enhanced understanding of the benefits. Immigrant women discussed fear in relation to their beliefs about breast cancer; however, they felt that the women's health session reconciled this fear because they came to understand that screening improved outcomes. For some women, they felt that going for routine health care and mammography provided greater hope for early treatment and survival.

*I used to believe that when women had breast cancer, they always died because there was no treatment for it, but after attending these (women's health) sessions, I learned that there are treatments and procedures and there are many women that have survived…always, there is hope to live.* (Focus group 2, Arabic, participant 2)

*I can find out the early warning signs of cancer and cure it early so that it does not get worse.* (FG 2, Vietnamese, participant 1)

*I learned many things from the health sessions… I came to know about breast cancer…I felt very afraid when I heard about breast cancer. If I go for the test and if there is any problem regarding cancer or any disease, then I'll be aware soon. I want to live; therefore, I go for a check-up.* (Focus group 1, South Asian, participant 4)

### 3.2. Theme 2: Social Support

This theme centered on distinct attributes of the peer health educator and the support she provided within the program, as well as other support from women of the community. Three subthemes reflected women's perception of support.

#### 3.2.1. Peer Educator Sense of Knowing

The peer health educator was perceived as caring about women's health. As a member of the same culture, she demonstrated this through knowing about women's sociocultural context, language, gender-related feelings of apprehension and worry related to screening, and information about accessing the health care system. Immigrant women felt the peer health educator was someone who listened and understood them, was engaged and respectful, and could be trusted with confidential information. Immigrant women were comfortable sharing health issues with the peer health educator not only because she was female but also because she conversed in their native language. This was important given that women were not necessarily proficient in the English language.

*The health educator was very respectful with our confidential information, and understood our issues.* (FG 3, Arabic, participant 5)

*Before, I didn't know where to get information about the health care system in Canada. The health educator program helped me a lot. So far, I have done two screenings…my personal experience is very pleasant.* (FG 1, Chinese, participant 8)

*There are some women that will not share their health issue with anyone…the program is a resource to find their solution….* (FG 1, South Asian, participant 2)

*Some of the ladies cannot speak English and they need an interpreter but there is a great facility in the health sessions for those ladies who do not speak English because the health educator speaks our language….* (FG 1, South Asian, participant 1)

#### 3.2.2. Being There and Fostering Cultural Safety

The WHEs accompaniment to screening was a valued aspect during the screening experience as she provided emotional and language support to immigrant women. Her presence provided a culturally safe environment to assist immigrant women to understand mammography; encourage them to take ownership of the experience; and help them to navigate the health care system. This was essential because it enabled immigrant women to have the procedure explained thoroughly and ask questions via interpretation and therefore understand the process of screening, findings, or need for follow-up.

*I feel more comfortable when I go with a woman like me and am able to speak the same language. For newcomers, it is very useful for them too, especially if you are shy to go to a male doctor to do the exam—the health educator helps you to book an appointment with a female doctor.* (FG 3, Arabic, participant 7)

*I felt more confident, safe, and comfortable with a health educator accompanying me to screening. I felt I understood my own health. There was more freedom to ask questions.* (FG 1, Vietnamese, participant 1)

#### 3.2.3. Woman to Woman Support

The benefits of the program were more than simple learning; rather participation involved gaining much needed social support for some immigrant women. Across all immigrant women groups, there was a sense that women were able to share experiences and views on health or social issues and support each other to have screening. Arabic immigrant women volunteered during women's health sessions and recruited newcomer women. They believed newcomers experienced greater difficulties of access due to family stressors, not knowing the settlement area, or a lack of knowledge of health or community services.

*Actually, I saw women in (women's health) sessions who were very close to each other and they were helping each other, especially newcomers. Also, I feel very happy when I see women attending these sessions and the program because they care about their health…I do really support women to attend.* (Interview 4, Arabic)

*During the session, women collaborate and mingle with each other and they get a chance to discuss their feelings.* (FG 2, South Asian, participant 2)

*…when we attended the health presentation (women's health sessions), we met with all ladies of our community and we shared our views with them. * (FG 1, South Asian, participant 4)

### 3.3. Theme 3: Screening Services Access for Women

This theme focused on immigrant women's perceptions of the peer health educator program as a whole and its connections to other health services. Three subthemes represent views on the program, including an unexpected finding of the role of family physicians.

#### 3.3.1. Valuing Immigrant Women's Health

All immigrant women groups appreciated that the government placed such importance on immigrant women's health through the creation of a peer health educator program. For some, immigrant women's health was not perceived to be a priority in their origin country. The peer health educator program was seen as accessible, focused on prevention, and delivered in a caring manner.

*As an Arabic woman, I found the government cares about my health and access to health services…I feel good because we are recognized and approached in our native language (through the program).* (Interview 1, Arabic)

*When I came to Canada, I was surprised about how much they care for women's health, because back home they do not care about women's health.* (FG 2, South Asian, participant 1)

*Since I have come to Canada, my mindset has totally changed and I think Canada is really people-oriented. It cares for people and it encourages people to care for themselves and their family. In Canada, the way of thinking is that you should prevent disease in pro-active way but in China, people only go to hospital when they are sick.* (Interview 1, Chinese)

*Thank you to the Canadian government for the opportunity to help the Vietnamese community.* (FG 2, Vietnamese, participant 1)

#### 3.3.2. Gender-Specific Supportive Screening Staff

While immigrant women were asked about their screening experience, they seemed to center on interactions with screening staff, such as nurses, mammography technicians, or administrative support staff. They believed screening staff were patient and the whole experience including the additional time required for language interpretation promoted a caring atmosphere. For some immigrant women, the female screening staff alleviated some anxiety and facilitated access to gender-specific information that would not have been possible with a male provider.

*Screening staff were all women, which was great; and all of them were friendly, kind and they gave us the comfort to do the tests, both mammogram and Pap test.* (FG 1, Arabic, participant 5)

*I noticed all the staff was good especially the nurse …she was very friendly and very helpful to me…I heard that she does the same for others too. * (FG 1, Arabic, participant 4)

*When I went for mammography screening center for the first time, I was confused and upset…the lady (nurse examiner) checked me very well. After my complete check-up, I was relaxed and happy.* (FG 1, South Asian, participant 4)

*Ladies feel reluctant to ask about sex information but when we meet lady doctors (nurse examiner or clinical nurse specialist) in the clinic, it's easier for us to ask any kind of information.* (FG 3, South Asian, participant)

#### 3.3.3. Family Physician Role

Emergent discussions about the role of the family physician were not anticipated; however, this topic represented an important issue for immigrant women when it came to access of screening. Arabic and Vietnamese immigrant women believed that the family physician was one of consultation if there was a breast lump found on exam and expressed confidence in them to investigate as needed. In contrast, Chinese and South Asian immigrant women believed that the family physician was responsible for providing information about breast cancer and screening. For Chinese immigrant women, they perceived that physicians treated illness and did not provide preventive health care, and the peer health educator program addressed this gap. Some South Asian immigrant women felt communication was a barrier that prohibited them from being able to ask questions about their health. If women had a male physician, they were reluctant to discuss female health issues and, for some, the physician did not provide breast cancer or screening information.

*It (health session) is very good. I know how to look for early warning signs, and can go see my doctor to cure it. * (FG 2, Vietnamese, participant 5)

*I wouldn't have paid so much attention to breast cancer if I hadn't been to this program. It would be already too late if my family doctor finds something….* (FG 1, Chinese, participant 2)

*You know in this country, we do not speak much English and my family doctor is a male; but for this program (and the educator), I would never know so much about breast cancer.* (FG 1, Chinese, participant 1)

### 3.4. Theme 4: Program Enhancements

Almost all immigrant women groups provided recommendations that built on already existing processes and functions established within the peer health educator program. The following subthemes focus on content, process, and dissemination.

#### 3.4.1. Content Revisions

Although the majority of Arabic and Chinese immigrant women felt information provided was highly valued and important, there were some immigrant women who felt that information was presented at a basic level of understanding, and, in turn, they felt it could have been more advanced. All immigrant women groups discussed the desire to learn more about other women's health topics, such as the effects of cholesterol, nutrition, colon cancer, osteoporosis, and how to deal with social and psychological stressors. Inviting physicians or other allied health to be guest speakers at women's health sessions was also recommended by Arabic immigrant women. Narrative stories of women diagnosed with early breast cancer to convey messages were proposed by some immigrant women as an effective means to learn the importance of breast screening. Arabic, Chinese, and South Asian immigrant women suggested using videos to reinforce and enrich health information provided by the peer health educator and to raise awareness about breast cancer and screening.

*It would be good to invite a doctor as a guest or professional to talk about new topics and answer our questions and our concerns, even if it is in English, we can have an interpreter.* (FG 3, Arabic, participant 2)

*Invite people who experienced cancer and had treatment and how they got back to their normal life, especially when cancer is discovered at early stages.* (FG 3, Arabic, participant 3)

*I think the video is complimentary to the health session. * (FG 1, Chinese, participant 7)

#### 3.4.2. Process Modifications

Arabic, Chinese, and South Asian immigrant women believed the process for holding women's health sessions could be improved through regular scheduling, more frequent sessions, and advanced notice. The rationale was to enable greater participation, keep information fresh and up-to-date, and allow women time to make plans around work, caregiver, or child care responsibilities. Advance notice of topics would also give women an opportunity to formulate questions prior to the session.

*I would suggest that the health educator prepares a monthly schedule for the sessions, and provides it to some women so they will know ahead of time about these sessions. Also, these women may help in bringing more new women to these sessions.* (FG 1, Arabic, participant 4)

*This program should be once a month and then easily we can manage to attend this program.* (FG 3, South Asian, participant 2)

#### 3.4.3. Greater Dissemination

Arabic and Chinese immigrant women felt that reaching out to newcomer immigrant women was important because the peer health educator program fostered a sense of inclusion to enable newcomer immigrant women to learn about health and the health care system and meet other women of their community. Arabic, Chinese, and South Asian immigrant women proposed additional methods of dissemination that included the distribution of translated flyers to other locations such as schools, churches, grocery stores, apartment buildings, and ethnic newspapers. Chinese immigrant women suggested expanding collaboration with other organizations with common goals of outreach to promote breast cancer screening to immigrant women.

*…newcomers still need help to find out about these programs and services.* (FG 3, Arabic, participant 4)

*I wish and encourage expanding the great volunteers to help women with the bus, to support and help newcomer women to also go to their appointments anywhere, or to go to the doctor, hospitals or clinics.* (FG 1, Arabic, participant 5)

*They should put flyers in banks, grocery stores, and in the buildings, and this advertisement should be in our language.* (FG 3, South Asian, participant 5)

*It would work best if the department who organizes the program can work together with family doctors, clinics or hospitals so that family doctors can remind people of doing the annual examination.* (Interview 5, Chinese)

## 4. Discussion

To our knowledge, this is among the first PAR projects to qualitatively examine multiple perspectives of immigrant women's experience with an existing peer health educator program situated within a public health unit in Canada. A key finding was that the women's health sessions provided encouragement for immigrant women to make lifestyle changes. This was important because sessions were presented holistically so that breast screening was imbedded within a framework of women's health and prevention. Similar findings have been reported in a program that utilized Latino lay health advisors to deliver health education sessions that promoted risk reduction and healthy lifestyle behaviours for cardiovascular health in the USA [15]. Primarily, female participants reported eliminating unhealthy foods and engaging in more daily physical activity as result of the program [15]. Also, a randomized controlled trial comparing a culturally adapted lay health educator led chronic disease self-management program to a wait list control group for Bangladeshi adults in the UK demonstrated an improvement in self-efficacy and self-care management behaviour in the intervention group [25].

The sense of responsibility for individual health maintenance was also a finding previously reported by South Asian immigrant women, whereby taking care of self was seen as holistic and included maintenance of diet and physical activity [26]. In another study, Vietnamese Canadian women believed that it was their responsibility to have a mammogram and Pap test, which included making an appointment for a regular check-up with the family physician [27]. The individual responsibility for seeking out health information or advice on disease prevention has also been reported among Australian women [28].

Knowledge and awareness about breast cancer screening provided a sense of hope and optimism for immigrant women that contradicted previously held beliefs of cancer as a disease that was not under individual control. In one pre- and postintervention study, a tailored series of publications to promote breast health and self-exam via a cultural newspaper was used and, after intervention, South Asian immigrant women were more hopeful with a breast cancer diagnosis and survival [29]. Furthermore, Smith and colleagues [30] examined memorable messages related to breast cancer and whether they evoked negative or positive emotions (i.e., hope) to determine which one motivated women (15% multiethnic) to engage in prevention and detection behaviours (i.e., mammography). While there was no difference in prevention and detection behaviours between groups, the authors speculated that women with positive emotions of hope may have believed that a breast cancer diagnosis was not something under their control [30]. Numerous studies have reported fatalism as a belief that addresses this perceived lack of control when it comes to breast cancer among immigrant women [7, 26, 31]. The perception that breast cancer is something that immigrant women cannot control may be related to a general lack of knowledge of the nature and course of breast cancer disease.

A key element of the program was the peer health educator role in the fact that she created a bridge between community and health services through distinct attributes including an understanding of the sociocultural context and needs of immigrant women. Similar findings were identified in a qualitative synthesis of studies focused on lay health advisor interventions for Hispanic/Latino populations [32]. The peer health educator's membership in the cultural community, caring, understanding of community needs and norms, and language skills were key attributes effective in promoting breast and cervical cancer screening [32]. Also, Southeast Asian women felt that community-based health navigators provided emotional support including physical presence, comfort, trust, and confidentiality in breast screening outreach [33].

The social environment created by the program not only facilitated access to breast health and screening but also provided mutual peer support and the creation of social networks. This is important because immigrant women settle in communities that are new and sometimes isolating due to loss of social support networks upon immigration and limited social engagement opportunities [34]. For some immigrant women, social networks and learning from other women about health promotion are preferred [26, 34].

The program introduced preventive health for the first time to some immigrant women, as it was not seen as important or prevalent in origin countries. Not having knowledge of preventive health care is common among immigrants residing in the USA and Canada [35, 36]. A tailored approach and coordinated services with screening centers ensure that immigrant women feel culturally safe during the screening experience. Factors that influenced satisfaction with the whole mammography experience among a diverse sample of women included ease of appointment scheduling, comfortable environment, the exam, personal factors (e.g., embarrassment, pain, and discomfort), mammography technologist care, receiving results, and follow-up [37]. A negative screening experience creates a barrier and a lack of desire to return for a repeat exam, a finding prevalent in a study examining African Americans' experience with their first mammography [38] and in another study exploring barriers to screening among black and minority ethnic groups in the USA [7].

For gender sensitive screening tests such as mammography, immigrant women prefer to be examined by a female health care provider, and this may also be related to why immigrant women in this study felt comfortable with screening staff, as they were all female. The cultural belief of modesty and potential examination by a male physician is a common barrier with female cancer screening exams [7, 31].

Some immigrant women groups believed that physicians were not proactive in providing information on breast cancer and screening. A family physician plays a vital role in breast cancer screening; yet, information or a screening recommendation is not always provided. A lack of physician recommendation was a barrier to mammography screening among Asian Indian women in the USA [8]. Immigrants settled in Canada for less than 10 years reported lower satisfaction with obtaining routine health exams and experienced greater challenges accessing health information from their family physician [39]. The family physician's role in facilitating access to screening has also been reported by Vietnamese Canadian women who believed that physicians were responsible for motivating and reminding them to have a mammogram and Pap test [27]. The role of the family physician is important to promoting mammography screening, an area where more work is needed to build on strengths of existing resources, and by capitalizing on public health programs already in existence.

Communication problems with family physicians related to health literacy continue to be a concern, at least for South Asian immigrant women. Conversational English ability does not necessarily mean that immigrant women can discuss health related concerns with their family physician as they may lack the English proficiency to express themselves especially when it relates to the use of medical terminology [40].

### 4.1. Program Enhancements and Public Health Actions

Public health actions were guided by immigrant women's perspectives from the* Program Enhancements* theme. The Vietnamese WHE left the program due to saturation of her population and changing demographics of immigrant settlement; therefore, modifications were targeted for the remaining three immigrant women groups.

To address the recommendation for advanced learning and expert guest speakers, partnerships were established within our public health unit and with other organizations and service providers (Table 3). With these collaborations, additional gender sensitive topics were provided. The use of stories and videos to promote breast cancer screening was an important strategy preferred by immigrant women. Therefore, we acted by incorporating story-telling in an online video series, whereby Arabic, Chinese, and South Asian immigrant women shared stories about their breast cancer screening experiences. Stories and narratives to promote breast cancer screening have been previously recommended by immigrant women [6, 31]. In particular, videos are a preferred method to deliver health promotion messages to immigrant women with limited English language proficiency [31, 41]. Videos have also been shown to improve screening intentions, knowledge, perceived risk for breast cancer, and perceived benefits of mammography among Chinese immigrant women [42].

Scheduling regular women's health sessions and providing advanced notice were important process changes that addressed immigrant women's busy lifestyles. Consequently, we partnered with a community center to provide monthly sessions for South Asian immigrant women and preplanned sessions with the other immigrant groups (Table 3). Work demands and multiple roles were factors mentioned in one qualitative study where immigrant women felt health information workshops were not held at times that were feasible for them [34]. Increasing the number of workshops was also a strategy to deal with women's busy lifestyles proposed by immigrant communities in a qualitative study in the USA [41].

The inclusion of newcomer outreach was another important recommendation that contradicted our previous understanding from settlement and immigration workers that newcomers had competing priorities of settlement and would not be interested in our program [43]. Subsequently, we employed additional dissemination and recruitment strategies to promote outreach to newcomer immigrant women that included expansion of collaborations with local primary health care teams (Table 3). Enhancing dissemination to newcomers and other immigrant women using community-based and ethnically focused media strategies (e.g., churches, grocery stores) was a finding in other studies among diverse immigrant groups [41, 44].

Enhancing collaboration and narrowing the gap between public health and primary care builds on existing strengths within each organization and, in turn, may reduce barriers and improve screening uptake in underscreened immigrant women [45]. To further expand collaboration and outreach, a partnership with a regional cancer center facilitated breast cancer screening promotion among the South Asian immigrant community. Continued partnerships with public health and the cancer center have resulted in other collaborative initiatives to promote screening.

Implementation of actions based on immigrant women's recommendations continued well after the project end date of January 2011 as the program to date continues to be funded by the public health unit and to enable access to cancer information and screening for immigrant women.

### 4.2. Strengths and Limitations

The strength of this PAR project was that it involved immigrant women as collaborative partners, provided insights of four diverse groups of immigrant women's experiences with a peer health educator program, and enabled immigrant women to be active in making changes to the program to meet their needs. These findings may be transferable to other settings where tailored peer health educator approaches are similarly provided for breast cancer screening promotion among Arabic, Chinese, South Asian, and Vietnamese populations.

There were some limitations in our study. While transferability of findings may be made to similar programs, the findings represent four immigrant women groups in one program in a midsized city in Canada and thus may not be transferable to some settings. Different organization of peer health educator approaches, variation in immigrant groups, sociocultural context of immigration, and the organization of health care services in other countries may differ from our program. We encountered challenges when recruiting participants from the program list such as the inability to locate participants due to mobility of immigrant women within or outside of the community or disconnected phone numbers. Additionally, the majority of women had accessed the program on more than one occasion reflecting the extent of engagement with the program; however, for those who had accessed the program early on, the elapsed time may have been an issue related to recall. Lastly, given that the program continued to be in existence and a source of continual support for some participants, they may have provided responses that were more desirable. However, the immigrant women who participated were assured that information they provided was confidential and that it would not influence their future participation in the program.

## 5. Conclusion 

This PAR project included immigrant women from Arabic, Chinese, South Asian, and Vietnamese communities. Involving immigrant women in public health promotion programming has been recommended in other studies [33, 34, 46] as it allows sociocultural context to be considered and the intended participants to be engaged in planning interventions. The four main themes provided insights into immigrant women's perceptions of the program including learning about breast health and prevention, access to social support, perceptions of screening and health services, and ways to improve programming. The relevance of findings is of value in understanding the sociocultural context of immigrant women, their information needs, and if barriers to access have changed since the implementation of the program. Only with this knowledge, can public health meet the changing needs of immigrant women communities. Findings are also relevant to public health programs that are interested in developing locally driven peer health educator programs or working collaboratively with peer health educators.

Future efforts will be required to improve collaborations between public health and primary care to narrow the gap in health disparities associated with cancer screening uptake among ethnic minority women. Further research could then serve to examine if these collaborations are effective at increasing mammography screening uptake among immigrant women.

## Figures and Tables

**Table 1:** Focus group characteristics.

Data collection	Arabic	Chinese	South Asian	Vietnamese
Focus groups	3-Arabic immigrant women only	1-Chinese immigrant women only	3-South Asian immigrant women only	3-Vietnamese immigrant women only

Participants	(1) *n* = 6	(1) *n* = 7	(1) *n* = 7	(1) *n* = 5
	(2) *n* = 8		(2) *n* = 6	(2) *n* = 4
	(3) *n* = 10		(3) *n* = 9	(3) *n* = 4

Language	Arabic	Mandarin	Urdu	Vietnamese

One-on-one interviews	*n* = 6	*n* = 6	*n* = 4	

Total	*n* = 30	*n* = 13	*n* = 26	*n* = 13

**Table 2:** Characteristics of immigrant women participants.

Participants	*n*	%	Language^*^	*n*	%
Arabic	30	36	English	17	18
South Asian	26	32	Arabic	27	28
Chinese	13	16	Assyrian	2	2
Vietnamese	13	16	Punjabi	17	18
Total	**82**	**100**	Urdu	10	10
			Hindi	2	2
			Chinese	11	11
			Vietnamese	13	13

Age	*n *	%	Country of origin	*n *	

40–49	29	35	China: 13	Syria: 4	
50–59	30	37	Vietnam: 13	Gaza: 3	
60–69	18	22	India: 14	Jordan, Kuwait	
70–74	5	6	Pakistan: 11	Damascus, Yemen: 4	
			Iraq: 11	Missing: 1	
			Sudan: 5		
			Palestine: 4		

Family doctor	%		Access to program	%	

Yes	76.83		Once	29.27	
No	8.00		Two times	10.98	
Missing	18.29		Three times or more	59.76	

^*^More than one selected.

**Table 3:** Participatory action research outcomes.

Program enhancement recommendations	Public health actions
*Content revisions* are as follows.	
Provide more advanced information with guest speakers: (i) invite doctors, specialist, or other health professionals (with interpreter to enable discussion).	Invited speakers to women's health session: public health nutritionist and registered dietician, physical activity specialist, tobacco cessation specialist, and settlement organization.

More topics related to women's health are as follows: (i) the effects of cholesterol; (ii) colon cancer; (iii) osteoporosis; (iv) social and psychological stress.	New topics covered include: (i) tobacco cessation; (ii) physical activity; (iii) colorectal cancer screening. Healthy eating series for newcomer immigrant women were established to learn about healthy food choices and integrate skill building through healthy snacks, label reading, and recipe sharing.

Use personal stories or narratives and videos: (i) to deliver content; (ii) to share stories of breast cancer screening experience or survivorship; (iii) to view videos on breast cancer screening in own home.	A series of videos using a story-telling format was developed within public health unit for each cultural group to share personal experiences of breast screening. Online search for culturally specific videos on different women's health topics was completed and catalogue of videos compiled to recommend to immigrant women.

*Process modifications* are as follows.	
Women's health sessions are not offered frequently enough and needs more regularity. Women recommended monthly prebooked sessions: (i) to reinforce messages using multiple efforts; (ii) to provide advanced notice in order to make plans for childcare or other caregiver responsibilities.	South Asian, Arabic, and Chinese: sessions were booked more regularly with advanced notification. Partnered with community organizations to provide monthly sessions in each community, and advertised early to allow women to make arrangements to attend.

*Dissemination strategies are as follows. *	
Expand outreach to include newcomers in other areas of the city by: (i) enlisting more volunteers; (ii) using other networks and collaborations for outreach; (iii) advertising in key areas, such as municipal housing. Use flyers to reach more newcomers including maps to session locations, such as: (i) grocery stores, mosques; (ii) apartment buildings; (iii) schools and churches.	The Arabic peer health educator engaged in outreach efforts specifically to newcomer women. Outreach for all groups has been expanded to include newcomers accessed through settlement and integration and primary health care contacts. Production of more small media in key languages to disseminate in new sites to reach more newcomers including the upgrade of online resources. Advertisement and word of mouth dissemination through settlement and integration centers.

Wider dissemination of the program in: (i) ethnic local newspapers; (ii) collaboration with primary health clinics, hospitals, or other prevention programs; (iii) online via website.	Media advertising in cultural newspapers has continued. Partnership with cancer center was established with South Asian peer health educator: (i) to expand promotion of the breast cancer screening in South Asian immigrant community; (ii) to develop new culturally appropriate small and mass media; (iii) to deliver education on South Asian culture to health professionals through lunch-time lectures at cancer clinic, screening clinics, and community agencies; (iv) to develop video for breast screening for South Asian women. Public health website provides up-to-date information on the program.

## Appendices

### A. Focus Groups Interview Guide

1. What has the experience of attending a peer health educator presentation (about breast health and breast screening) meant to you?
2. What benefits do you feel you received from attending the presentation given by the peer health educator?
3. Were there things that made it challenging/difficult to attend the Women's Health presentation? If so, what was challenging/difficult?
4. Describe strengths of the peer health educator program.
5. Describe areas of improvement for the peer health educator program? How can we make it better?

If Focus Group members attended OBSP:

1. What were the key/main things that influenced your decision to attend breast screening?
2. How did you feel having a peer health educator accompany you to breast screening?
3. As an immigrant woman, what was good (a strength/helpful) about the experience you had at the screening program?
4. As an immigrant woman, how could the screening experience be improved?

### B. One-on-One Interview Guide

1. Tell me about your experience attending a Women's Health presentation.
2. Explain to me how these presentations affected you in terms of what you learned or gained personally from participating.
3. Tell me a little bit about your experience attending the screening.
4. Tell me about the parts of the peer educator program that really helped you.
5. Tell me what we could do to make this program better for women like you.

If participants attended OBSP, ask the following questions:

1. What influenced your decision to attend breast screening?
2. Did a peer health educator accompany you to breast screening? If so, how did you feel?
3. As an immigrant woman, what was good about the experience you had at the screening program?
4. What ways do you think the screening experience could be improved?
